# Plants and Associated Soil Microbiota Cooperatively Suppress Plant-Parasitic Nematodes

**DOI:** 10.3389/fmicb.2020.00313

**Published:** 2020-02-28

**Authors:** Olivera Topalović, Muzammil Hussain, Holger Heuer

**Affiliations:** ^1^Institute for Epidemiology and Pathogen Diagnostics, Julius Kühn-Institut, Federal Research Centre for Cultivated Plants, Braunschweig, Germany; ^2^State Key Laboratory of Mycology, Institute of Microbiology, Chinese Academy of Sciences, Chaoyang, China

**Keywords:** disease-suppressive soils, plant-parasitic nematodes, root exudates, rhizosphere microbiome, root endophytes, nematode antagonists, induced systemic resistance

## Abstract

Disease suppressive soils with specific suppression of soil-borne pathogens and parasites have been long studied and are most often of microbiological origin. As for the plant-parasitic nematodes (PPN), which represent a huge threat to agricultural crops and which successfully defy many conventional control methods, soil progression from conducive to suppressive state is accompanied by the enrichment of specific antagonistic microbial consortia. However, a few microbial groups have come to the fore in diminishing PPN in disease suppressive soils using culture-dependent methods. Studies with cultured strains resulted in understanding the mechanisms by which nematodes are antagonized by microorganisms. Recent culture-independent studies on the microbiome associated with soil, plant roots, and PPN contributed to a better understanding of the functional potential of disease suppressive microbial cohort. Plant root exudation is an important pathway determining host-microbe communication and plays a key role in selection and enrichment of a specific set of microbial antagonists in the rhizosphere as first line of defense against crop pathogens or parasites. Root exudates comprising primary metabolites such as amino acids, sugars, organic acids, and secondary metabolites can also cause modifications in the nematode surface and subsequently affect microbial attachment. A positive interaction between hosts and their beneficial root microbiota is correlated with a low nematode performance on the host. In this review, we first summarized the historical records of nematode-suppressive soils and then focused on more recent studies in this aspect, emphasizing the advances in studying nematode-microbe interactions over time. We highlighted nematode biocontrol mechanisms, especially parasitism, induced systemic resistance, and volatile organic compounds using microbial consortia, or bacterial strains of the genera *Pasteuria*, *Bacillus*, *Pseudomonas*, *Rhizobium*, *Streptomyces*, *Arthrobacter*, and *Variovorax*, or fungal isolates of *Pochonia*, *Dactylella*, *Nematophthora*, *Purpureocillium*, *Trichoderma*, *Hirsutella*, *Arthrobotrys*, and *Mortierella*. We discussed the importance of root exudates in plant communication with PPN and soil microorganisms, emphasizing their role in microbial attachment to the nematode surface and subsequent events of nematode parasitism. Comprehensive understanding of the plant-beneficial microbial consortia and the mechanisms underlying disease suppression may help to develop synthetic microbial communities for biocontrol of PPN, thereby reducing nematicides and fertilizers inputs.

## Introduction

Disease suppressive soils are the conspicuous prototype of a microbe-mediated plant defense against pathogen infection. In general, suppressive soils are those in which soil-borne pathogens and parasites do not establish or persist, establish but cause limited or no disease at all, or establish and cause disease onset initially before it abates (Baker and Cook, 1974; Weller et al., 2002). By contrast, agricultural soils in which pathogens and parasites infect plants and cause diseases are referred to as non-suppressive or conducive soils (Weller et al., 2002; Garbeva et al., 2004). By definition, general soil suppressiveness, a typical epitome of cumulative soil microbiome competitive activities, is supposed to act against a wide range of soil-borne diseases (Cook and Baker, 1983; Borneman and Becker, 2007). These soils operate on the principle of “seed, feed, and weed” such as it could be initiated by the addition of organic matter in the soil or on the presence of seed and root exudates (seed), which results in the uptake of nutrients by diverse microorganisms (feed), and consequently limiting the outbreak of pathogens and parasites (weed). Therefore, general soil suppressiveness is reduced by soil steaming, but cannot be transferred by small amounts of suppressive soil to conducive soil (Cook and Baker, 1983; Weller et al., 2002). On the other hand, specific soil suppressiveness is typically induced in field soils during crop monoculture after a disease outbreak. It relies on the antagonistic activities of specifically enriched microbial consortia that disrupt the life cycle of plant pathogens or parasites (Raaijmakers and Mazzola, 2016). Specific soil suppressiveness can be eliminated by soil sterilization and biocide treatments and is transferable to a conducive soil with small amounts of a suppressive soil (0.1–10%) (Yin et al., 2003a; Borneman and Becker, 2007). The criterion of transferability implies that the transferred antagonistic microbes can sufficiently multiply in the conducive soil to reach suppressive densities. This depends on interactions with soil biota, roots, or for specialized antagonists on the density of the pathogen. The distinction between general and specific soil suppressiveness may thus be rather related to the diversity of antagonists than the mechanisms of suppressiveness.

**FIGURE 1:**
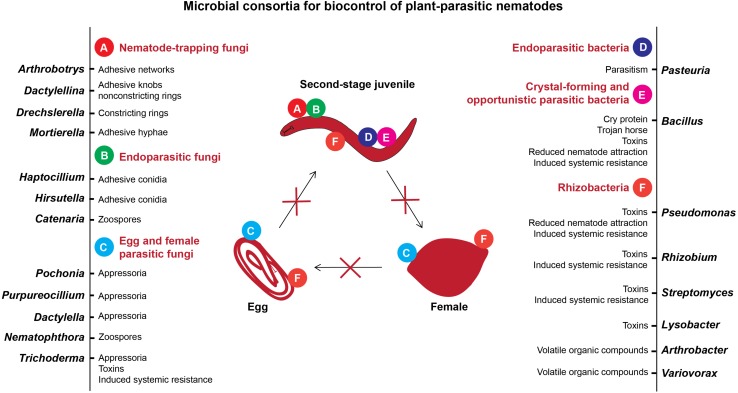
Microbial antagonists of plant-parasitic nematodes and mechanisms of their antagonism.

Soils with specific suppressiveness have been reported for plant-parasitic nematodes (PPN) from distinct geographical locations worldwide. Importantly, the life cycle of PPN is distinct from those of pathogenic fungi and bacteria, and diverse microbes were characterized to prey and parasitize different stages of nematodes (Li et al., 2015). Consequently, most of the studies were mainly focused on identifying the microbes that can directly kill PPN, while the plant-mediated microbial suppression of nematodes was often overlooked. In soil systems, nematophagous fungi and bacteria have diverse strategies to attack PPN, e.g., nematode-trapping fungi form adhesive hyphal traps, endoparasitic fungi and bacteria use spores, egg- and female-parasitizing fungi use hyphal tips, and several fungi and bacteria produce toxins to prevent plant roots from nematode invasion (Figure 1). Recently, using the next-generation technologies several studies have come to the fore signifying the role of soil and root microbiota that disturb the performance of PPN. Thus, a detailed understanding of the microbiome associated with the soil, plant roots, and distinct life stages of PPN may enable us to engineer (synthetic) core microbial consortia that can act as a sustainable alternative to control nematode diseases and to enhance crop productivity. Herein, we will review the studies highlighting the contribution of microbes in inhibiting nematodes in disease suppressive soils using culture-dependent and culture-independent methods. In addition, we will highlight the importance of root-associated microbiota in plant-nematode interactions. Finally, we will tackle some important points on the role of root exudates in nematode infection and their effects on the nematode surface coat (SC), which influence nematode-microbe interactions in soil. This review will give insights into the plant-nematode-microbe interactions in suppressive soils.

## History of Nematode-Suppressive Soils

The chronological records of nematode-suppressive soils reliably show the progress that has been made in understanding nematode-microbe interactions. The decline of PPN populations in field soils was first reported in 1962 by Collingwood for the cyst nematode *Heterodera avenae* in the United Kingdom under intensive cropping of cereal hosts (Collingwood, 1962; Kerry, 1982). Since then, suppressive soils have been reported for several nematode species including *H. avenae* in the United Kingdom (Gair et al., 1969; Williams, 1969; Kerry, 1975), *Heterodera schachtii* in Netherlands, United Kingdom, and United States (Heijbroek, 1983; Crump and Kerry, 1987; Westphal and Becker, 1999), *Globodera rostochiensis* in Germany (Roessner, 1987), *Globodera pallida* in the United Kingdom and Germany (Crump and Flynn, 1995; Eberlein et al., 2016), *Heterodera glycines* in the United States and China (Chen et al., 1996b; Sun and Liu, 2000; Hamid et al., 2017; Hussain et al., 2018), the false root-knot nematode *Nacobbus aberrans* in Mexico (Zuckerman et al., 1989), root-knot nematodes *Meloidogyne* spp. in Mexico, United States, Spain, and Germany (Bird and Brisbane, 1988; Zuckerman et al., 1989; Chen et al., 1994b; Pyrowolakis et al., 2002; Bent et al., 2008; Adam et al., 2014b; Giné et al., 2016), and the ring nematode *Mesocriconema xenoplax* in the United States (Kluepfel et al., 1993, 2002).

General and specific suppression of soil-borne pathogens and parasites is most often of microbiological origin. In 1960s, the formalin application was the only factor known to reduce soil suppressiveness against *H. avenae* in agricultural fields (Williams, 1969). It was suggested that the application of formalin inhibited the parasitic fungi *Nematophthora gynophila* and *Pochonia chlamydosporia* and thereby resulted in an increased population density of *H. avenae* (Kerry et al., 1980; Kerry, 1988). Later, several studies demonstrated the microbial involvement in soil suppressiveness by soil autoclaving or heating (Bird and Brisbane, 1988; Zuckerman et al., 1989; Kluepfel et al., 1993; Chen et al., 1996b; Weibelzahl-Fulton et al., 1996; Westphal and Becker, 1999; Sun and Liu, 2000; Bent et al., 2008; Adam et al., 2014b; Eberlein et al., 2016; Giné et al., 2016; Hamid et al., 2017; Bhuiyan et al., 2018), by the application of biocides (Kerry et al., 1980; Crump and Kerry, 1987; Westphal and Becker, 1999; Sun and Liu, 2000; Westphal and Becker, 2001; Pyrowolakis et al., 2002; Yin et al., 2003a, b; Bent et al., 2008; Song et al., 2017), and through soil transplantation (Mankau, 1975; Stirling and Kerry, 1983; Kluepfel et al., 1993; Westphal and Becker, 2000; Sun and Liu, 2000; Yin et al., 2003a, b; Chen, 2007; Bent et al., 2008). Notably, the soil suppressiveness was also observed to be transferred by egg-suspensions of the root-knot nematode *Meloidogyne incognita* (Orion et al., 2001) and cysts of the sugar beet cyst nematode *H. schachtii* (Westphal and Becker, 2001). Some soils with specific suppressiveness have been characterized and underlying microbial mechanisms were proposed, including parasitism and antibiosis. The role of parasitism in nematode-suppressive soils has extensively been studied for root-knot nematodes (Mankau, 1975), cereal cyst nematodes (Stirling and Kerry, 1983), sugar beet cyst nematodes (Olatinwo et al., 2006b; Becker et al., 2013), and soybean cyst nematodes (Chen, 2007). Antibiosis is an important mechanism of biocontrol, in which the antagonist produces metabolites, such as lytic enzymes, toxins, antibiotics, or volatile compounds, which potentially disrupt the pathogen invasion. Unlike the essential increased initial concentration of parasites for a successful nematode control, it has been noted that nematode suppression by some rhizobacteria, employing different modes of action including antibiosis, can be accomplished at lower microbial densities (Kluepfel et al., 1993). This was the case with ring nematode (*M. xenoplax*)-suppressive soils, where *Pseudomonas* sp. producing salicylic acid were found to dramatically alter the development of eggs and to be involved in the inhibition of egg hatch (Kluepfel et al., 2002).

More and more studies on soil microorganisms associated with nematode decline have unraveled an array of new microbial species with an antagonistic potential against PPN. In the following lines we will review bacteria and fungi that were either isolated from the diseased or dead nematodes, or that were identified in nematode-suppressive soils by next-generation sequencing. The culture-dependent and culture-independent approaches in determining nematode antagonists will be compared and discussed.

## Culture-Dependent Studies on Microbiota in Nematode-Suppressive Soils

As specific soil suppressiveness is determined by the activity of certain antagonistic microbial species that are main culprits for the decrease in nematode population density, the cultivation of such organisms on growth media is an essential step for their large-scale multiplication and prolonged utilization in nematode control. As discussed in the next section, many studies using culture-independent approaches unraveled a high diversity of the microbiome associated with PPN in suppressive soils. However, it has been proposed that only 1% of microorganisms in nature are cultivable (Amann et al., 1995). This significantly decreases the range of nematode antagonists that can be mass-produced. Moreover, cultivation of nematode obligate parasites represents an additional challenge since it often requires the presence of the nematode host. Antagonistic effects of diverse microorganisms were detected in *in vitro* studies on the biology of free-living nematodes (Huang et al., 2005; Rae et al., 2008), or they appeared as by-products of studies on soil suppressiveness against other pathogens and parasites of plants (Adam et al., 2014a). Nevertheless, there are still numerous microbial species that were isolated from nematodes in suppressive soils (Bird and Brisbane, 1988; Chen et al., 1996a; Weibelzahl-Fulton et al., 1996; Borneman et al., 2004; Borneman and Becker, 2007; Chen, 2007; Adam et al., 2014b; Eberlein et al., 2016; Giné et al., 2013, 2016). In this review we focused on the microorganisms associated with the most important group of PPN, sedentary endoparasites (root-knot and cyst nematodes). We assigned these microbes to two different groups: (1) microbial species associated with suppression of migratory stages of sedentary endoparasitic nematodes and (2) microbial species associated with suppression of sedentary stages of sedentary endoparasitic nematodes.

### Microbial Species Associated With Suppression of Migratory Stages of Sedentary Endoparasitic Nematodes

The most studied microorganism associated with migratory stages of PPN in suppressive soil is the bacterium *Pasteuria* (Mankau, 1975). The obligate nature of *Pasteuria* spp. makes them a promising candidate for biocontrol of PPN. Several species of *Pasteuria* have been reported to parasitize nematodes. *Pasteuria penetrans* parasitizes *Meloidogyne* spp. (root-knot nematodes), *Pasteuria nishizawae* parasitizes *Globodera* spp. and *Heterodera* spp. (cyst nematodes), *Pasteuria thornei* parasitizes *Pratylenchus* spp. (root-lesion nematodes), and *Pasteuria usgae* parasitizes *Belonolaimus* spp., a sting nematodes (Preston et al., 2003; Noel et al., 2005). The attachment of *Pasteuria* spp. endospores to the nematode cuticle is a first step during parasitism. In case of *P. penetrans* parasitizing *Meloidogyne*, the spores first attach to the infective second-stage juveniles (J2) in soil, and then, as J2 enter the roots, bacteria produce microcolonies inside the nematode’s pseudocelom. Eventually, the development of eggs within females is disrupted (Li et al., 2015). However, in other PPN, endospores can germinate through the cuticle and complete the bacterial life-cycle with new endospores forming in the nematode juvenile (e.g., *Pasteuria* on *H. avenae*) (Davies et al., 1990). *Pasteuria* spp. were repeatedly isolated from soils that exhibited suppressiveness against different nematode species (Stirling and Wachtel, 1980; Stirling and White, 1982; Bird and Brisbane, 1988; Chen et al., 1994b; Weibelzahl-Fulton et al., 1996; Stirling et al., 2017; Bhuiyan et al., 2018). However, the presence of *Pasteuria* endospores in soil does not always guarantee nematode parasitism and small numbers of attached spores may not lead to the infection of PPN. For instance, Stirling et al. (2017) have detected *Pasteuria* spp. in 56% of the sugarcane fields, but only 5% of the observed nematodes had attached spores. In one field, *Pasteuria* sp. was suggested to be responsible for the suppression of *Meloidogyne javanica*, but only in case when the inoculated J2 had to cross a distance of more than 4 cm to reach the roots. The low parasitism rate in some cases appears as a result of a very high nematode-bacterium specificity (Chen and Dickson, 1998). Concentration of bacterial endospores in soil and persistence of antagonism were tested by Bhuiyan et al. (2018). It was shown that reproduction of *M. javanica* was reduced in a concentration-dependent manner, and the level of parasitism was very high with a 96% decrease in reproduction at 6 months post inoculation, to a 81% decrease in reproduction at 20 months post inoculation. Amongst other microbial antagonists isolated from suppressive soils that showed high antagonistic effects against PPN, *Streptomyces costaricanus* was effective against a wide range of nematode species (Dicklow et al., 1993; Esnard et al., 1995). The fungus *Hirsutella* spp. was demonstrated to produce conidia that adhere to the cuticle of J2 of cyst nematodes, and thereafter penetrate, digest and kill the nematode before root invasion (Stirling and Kerry, 1983; Hussain et al., 2016; Wang et al., 2016a, b). Topalović et al. (2019) have isolated several bacterial genera from the cuticle of the root-knot nematode *Meloidogyne hapla* in soils with a varying degree of suppressiveness. These were assigned to *Microbacterium*, *Sphingopyxis*, *Brevundimonas*, *Acinetobacter*, and *Micrococcus*. Although these are bacteria not obligate parasites of PPN, they showed antagonistic effects against J2 by increasing mortality, reducing motility, or reducing J2 invasion into the roots. One of the strains reduced hatching of J2 from the eggs.

While a direct antagonism of microbes to PPN was mostly regarded as the main mechanism of soil suppressiveness, more recently the plant is understood as a holobiont in association with its microbiome (Hassani et al., 2018). Using split-root systems of tomato plants, Adam et al. (2014a) showed that for *Bacillus* isolates, although selected as potential biocontrol strains based on their ability to produce nematicidal and fungicidal compounds, the main mechanism suppressing root-knot nematodes was not direct but plant-mediated. Microorganisms do not only target mobile stages of PPN in soil, but can also colonize the roots to parasitize the sedentary stages of endoparasitic nematodes, which is brought up in the following lines.

### Microbial Species Associated With Suppression of Sedentary Stages of Endoparasitic Nematodes

Amongst the first isolated microorganisms that exhibited parasitism against sedentary stages of PPN were the fungi *N. gynophila* and *P. chlamydosporia* (former *Verticillium chlamydosporium*) that lead to the decline of the cyst nematode *H. avenae* (Kerry, 1982; Stirling and Kerry, 1983). *P. chlamydosporia* is a saprophytic fungus in soil whose association to nematode eggs and cysts in soils is common (Giné et al., 2016; Song et al., 2017; Hu et al., 2018, 2020). Suppression of *H. schachtii* in Californian soil has been extensively studied, and transfers of amounts as small as 0.1% of this soil to a conducive soil have resulted in a significant decline of nematode reproduction (Westphal and Becker, 2000). Soil suppressiveness was correlated with a high presence of the fungi *Dactylella oviparasitica* and *Fusarium oxysporum* in nematode cysts and eggs (Westphal and Becker, 2001). However, involvement of *F. oxysporum* in nematode suppression is still speculative since in several subsequent studies it failed to cause a nematode decline (Olatinwo et al., 2006a, b; Gao et al., 2008; Becker et al., 2013). In addition, Becker et al. (2013) have pointed out that, although *D. oviparasitica* was highly effective in egg parasitism, viable eggs still remained resistant to this fungus. Since it is also able to parasitize J2, they proposed J2 as a target when applying *D. oviparasitica* as a biocontrol agent against cyst nematodes. Egg parasitism by certain strains of *Pseudomonas* and *Bacillus* species isolated from suppressive soils is also important to mention in case of ectoparasitic PPN (Westcott and Kluepfel, 1993; Kluepfel et al., 2002; Colagiero et al., 2017). Kluepfel et al. (2002) have detected several candidate genes in an isolated *Pseudomonas* strain to be responsible for egg toxicity.

Consortia of other fungi were also isolated from eggs and females of root-knot or cyst nematodes in several studies and for some of them antagonistic effects were confirmed in greenhouse assays (Marban-Mendoza et al., 1992; Chen et al., 1994a, 1996a; Crump and Flynn, 1995). Using microbial strains isolated from diseased nematode stages, different mechanisms to antagonize the nematodes were shown, such as parasitism, production of toxins and traps, or plant-mediated mechanisms. The mass production of antagonists is preceded by their isolation, thus a culture-dependent approach to study nematode-suppressive microbes is of enormous importance. However, microbial identification using DNA-based methods gives a more accurate representation of microbial consortia associated with suppression of PPN, and the following sections cover studies in this aspect.

## Culture-Independent Studies on Microbiota Associated With Ppn in Nematode-Suppressive Soils

Although several potential microbial antagonists are known to regulate nematode population densities under laboratory and controlled greenhouse conditions, most of them failed to antagonize PPN in the field environment at a distinct geographical location. This inconsistency of microbes to fully express their antagonistic characteristics have been attributed to the lack of their survival within complex microbial communities in soil, or to their inability to better colonize plant roots under different environmental conditions (Alabouvette et al., 2009). Besides, soil suppressiveness is established because of the activity of microbial consortia rather than of just a single species (Costa et al., 2012; Raaijmakers and Mazzola, 2016). Therefore, several cultivation-independent approaches, including community profiling by oligonucleotide fingerprinting of rRNA genes (OFRG), denaturing gradient gel electrophoresis (DGGE), and high-throughput sequencing of PCR amplified taxonomic marker genes have been employed to decipher microbial cohort contributing to disease suppressiveness within the complex microbial communities, and to infer microbial antagonistic activities operating in nematode-suppressive soils.

### Microbes Identified in a Direct Association to PPN

Several studies have focused on the structure and diversity of microbial communities associated with different life stages of PPN. Since nematode mobile stages reside in soil until they find the suitable roots for feeding, their association with soil microorganisms has been studied the most. A microbial community analysis of root-knot nematode infective J2 revealed a dominance of the bacterial genera *Sphingomonas*, *Micrococcus*, *Bacillus*, *Methylobacterium*, *Rhizobium*, and *Bosea*, and of the fungal genera *Davidiella* and *Rhizophydium* in soils that were suppressive against *M. hapla* (Adam et al., 2014b). Also, J2 of *M. incognita* were enriched by fungi of the genera *Malassezia*, *Plectosphaerella*, *Gibellulopsis*, and *Lectera* in a soil suppressive against this nematode species (Elhady et al., 2017). Notably, Elhady et al. (2017) also found an association of the bacterium *Bacillus thuringiensis* and the fungus *Plectosphaerella cucumerina* with *M. incognita* J2 in soil. *B. thuringiensis* produces proteinaceous protoxin crystals (called crystal protein or Cry protein) that cause lysis of the intestine and the nematode’s death (Griffitts et al., 2005; Vachon et al., 2012). *P. cucumerina* was also isolated from the egg masses of *M. incognita* (Yu and Coosemans, 1998) and eggs of *G. pallida* (Kooliyottil et al., 2017), and has been found to infect eggs of *G. rostochiensis* (Atkins et al., 2003). The microbial community analysis using bacterial 16S rRNA gene and ITS amplicon sequencing from *G. pallida* females showed a dominance of diverse microbiota such as *Burkholderia*, *Bosea*, *Rhizobium*, *Devosia*, *Ralstonia*, and *Streptomyces*, and the fungal genera *Davidiella*, *Hirsutella*, *Malassezia*, *Microdochium*, *Monographella*, and *Penicillium* in potato monoculture soil (Eberlein et al., 2016). Some of the bacterial genera have been previously associated with infective stages of root-knot and lesion nematodes in suppressive soils (Adam et al., 2014b; Elhady et al., 2017).

The adult females of cyst nematodes first appear on the roots and finally develop into mature brown cysts which end up in the surrounding soil. The eggs inside the cysts are colonized by a number of microbes (Nour et al., 2003), and it has been reported that the cysts can transfer suppressive microbes into a nematode-conducive soil (Westphal and Becker, 2001). The earliest effort to characterize bacterial and fungal microbiota inhabiting the cysts in suppressive soil using OFRG identified major taxonomic groups of bacteria (α-*Proteobacteria*, β-*Proteobacteria*, γ-*Proteobacteria*, *Cytophaga-Flexibacter-Bacteroides*, and *Actinobacteria*) and fungi (*D. oviparasitica*, *F. oxysporum*, and *Lycoperdon* spp.), and were then coupled with quantitative PCR to highlight the association of *Rhizobium*- and *D. oviparasitica*-like rDNA groups with the suppressiveness against *H. schachtii* (Yin et al., 2003a, b). In addition, using high-throughput amplicon sequencing to examine fungal and bacterial communities from cysts in soil suppressive to *H. glycines*, Hu et al. (2017) demonstrated that suppressiveness is associated with the change in relative abundance of diverse bacterial and fungal taxa, especially *P. chlamydosporia*, *Exophiala* spp., and *Clonostachys rosea*. Similarly, the investigation of fungi from egg masses of *M. incognita* revealed a total of 11 phylotypes, including *P. chlamydosporia*, that were proposed to be involved in regulating root-knot nematode populations (Bent et al., 2008). Recently, Hussain et al. (2018) highlighted the interaction between *H. glycines* cysts and soybean root microbiota using high-throughput sequencing of the V4 region of the bacterial 16S rRNA gene. Specifically, they showed that the microbial consortia enriched upon nematode infection in the rhizosphere and root endosphere also colonized nematode cysts. This points out that cysts are able to serve as an inoculum source for nematode suppressiveness. Moreover, the *H. glycines* cyst bacterial community was established by the consecutive selection of bacterial taxa from the root endosphere. Bacterial microbiota including, *Chitinophaga*, *Yersinia*, *Lentzea*, *Niastella*, and *Pseudoxanthomonas* were dominating the cyst bacterial community in suppressive soil (Hussain et al., 2018). *Chitinophaga* is a chitin-decomposer with chitinase activity (Sangkhobol and Skerman, 1981; McKee et al., 2019) and the chitin is an essential element of the nematode eggshell (Gortari and Hours, 2008). Chitinases could have an effect on egg viability and hatching (Chen and Peng, 2019). Further, the chitinase-generated chitooligosaccharides could induce the expression of parasitism-related genes in egg-parasitizing fungi (Escudero et al., 2016) inhabiting the cysts (Hu et al., 2018, 2020). Thus, microbiota directly associated with PPN stages in suppressive soils showcasing the ability of nematode suppression elucidate the potential role of diverse microbial consortia in this enticing microbiological milieu.

### Microbes Detected in Soils With a Reduced Nematode Performance

The recent use of cultivation-independent technologies has provided deep insights into the microbial community composition of rhizosphere soils and their contribution in the suppression of PPN. Diverse bacterial and fungal taxa were reported to be enriched in soils with a low presence of PPN or with a poor PPN performance on the host plants grown in these soils. For instance, Giné et al. (2016) investigated the structure of bulk soil microbiota using bacterial and fungal community fingerprinting by DGGE. They found that the bacteria assigned to *Lysobacter*, *Flavobacterium*, *Chryseobacterium*, *Flexibacter*, *Steroidobacter*, and *Methylobacterium*, and the fungi assigned to *Cladosporium*, *Fusarium*, *Mortierella*, *Preussia*, and *Stachybotrys* were frequently detected in soils with a low record of root-knot nematodes. Consequently, the fungus *P. chlamydosporia* was isolated and verified to parasitize *Meloidogyne* eggs (Giné et al., 2016). In another study, *P. chlamydosporia*, together with the fungus *Purpureocillium lilacinum* and the bacterium *Pseudomonas* sp., were also marked as major taxa inhabiting the rhizosphere of soybean plants that were grown in soils from north-eastern China, and that were suppressive against *H. glycines* (Hamid et al., 2017). These two egg-parasitic fungi showed a strong geographical preference. The bacterium *Pseudomonas* sp. had a higher relative abundance in the suppressive than in the conducive soils across all geographical locations (Hamid et al., 2017). It was hypothesized that soils with the ability to suppress specific diseases have a memory of previously encountered pathogens (Raaijmakers and Mazzola, 2016). Those microbial consortia that responded to pathogen attack in a previous plant generation are probably the key drivers of soil memory against specific pathogens (Berendsen et al., 2018). Thus, plants can adopt a “cry for help” strategy during pathogen invasion, leading to the selective enrichment of a specific set of microbes in the soil (Bakker et al., 2018). Furthermore, the high-throughput sequences of the fungal ITS locus showed that both *P. chlamydosporia* and *P. lilacinum* were increased in the rhizosphere upon combined soil application of the nematode endoparasitic fungus *Hirsutella minnesotensis* and chitosan to suppress *H*. *glycines* (Mwaheb et al., 2017). Similarly, soils with low population densities of *Pratylenchus neglectus* and *Meloidogyne chitwoodi* were enriched by the rhizobacteria *Arthrobacter*, *Bacillus*, and *Lysobacter* as revealed by high-throughput sequence analysis of the V4 region of the bacterial 16S rRNA gene (Castillo et al., 2017). With regards to the bacteria, Hussain et al. (2018) using high-throughput sequencing of the V4 region of the bacterial 16S rRNA gene identified less than 30 genera, including *Pasteuria, Pseudomonas*, and *Rhizobium*, that were enriched in the rhizosphere and/or root endosphere of soybean grown in suppressive soil, and associated with the suppression of *H. glycines* under a long-term crop monoculture.

Importantly, we summarized the nematode biocontrol mechanisms of the most commonly detected antagonistic microbial consortia from suppressive soils such as the fungi *Pochonia*, *Dactylella*, *Nematophthora*, *Purpureocillium*, *Trichoderma*, *Hirsutella*, *Haptocillium*, *Catenaria*, *Arthrobotrys*, *Dactylellina*, *Drechslerella*, and *Mortierella*, and the bacteria *Pasteuria*, *Bacillus*, *Pseudomonas*, *Rhizobium*, *Streptomyces*, *Arthrobacter*, *Lysobacter*, and *Variovorax* (Figure 1). With respect to the fungi, a wide range of the antagonistic species has been described with the ability to trap, parasitize, or intoxicate nematodes, or suppress endoparasitic PPN by inducing systemic resistance in plants (Yang et al., 2007; Szabó et al., 2012; Askary, 2015; Martínez-Medina et al., 2017; Zhang et al., 2019). Likewise, bacterial antagonists often have more than one mode of action. Although the parasitism of nematodes by *Pasteuria* has been the most extensively studied, the efficiency of nematode antagonism by bacterial toxins, production of volatile organic compounds and nematode repellence from the roots by bacterially induced systemic resistance in plants has also been studied (Siddiqui and Mahmood, 1999; Wei et al., 2003; Sikora et al., 2007; Tian et al., 2007; Li et al., 2015). The results of recent studies on characterization of microbiota associated with soil, plant roots, and nematodes in suppressive soils are summarized in Table 1.

**TABLE 1:** DNA-based characterization of the bacterial and fungal microbiota inhabiting the soil, plant roots, and nematode stages (mobile and sedentary) in disease suppressive soils.

Nematode (Reference)	Microhabitat	Technique	Microbial genera/species
*Meloidogyne hapla* (Adam et al., 2014b)	J2/Bulk soil	ITS – DGGE	*Malassezia, Aspergillus, Cryptococcus, Chaetomium, Eurotium, Ganoderma, Cladosporium, Davidiella, Mortierella, Cylindrocarpon, Rhizophydium*
		16S – DGGE	*Bradyrhizobium, Sphingomonas, Staphylococcus, Micrococcus, Bacillus, Propionibacterium, Methylobacterium, Streptococcus, Solirubrobacter, Janthinobacterium, Rhizobium, Pedomicrobium, Ochrobactrum, Nitrospira, Devosia, Kaistia, Magnetospirillum, Bosea, Rhodobacter, Pseudomonas*
		16S – amplicon	*Micrococcus, Rothia, Geobacillus, Streptococcus, Anaerococcus, Peptoniphilus, Clostridium, Mycoplasma, Ochrobactrum, Hirschia, Haematobacter, Paracoccus, Malikia, Janthinobacterium, Neisseria, Vogesella, Shigella, Acinetobacter, Acinetobacter, Enhydrobacter, Pseudomonas*
*Meloidogyne incognita Pratylenchus penetrans* (Elhady et al., 2017)	Infective stage/bulk soil	16S – DGGE	*Burkholderia, Fusicatenibacter, Burkholderia, Oscillatoria, Curvibacter, Acinetobacter*
		16S – amplicon	*Paraburkholderia, Ralstonia, Streptococcus, Staphylococccus, Bacillus thuringiensis, Streptococcus, Acinetobacter, Gemmatimonas, Anaerococcus, Pelomonas, Burkholderia, Neorhizobium*
		ITS – amplicon	*Plectosphaerella, Penicillium, Lectera, Tetracladium, Chaetomium, Petriella, Malassezia, Taphrina, Alternaria, Stemphylium, Cladosporium, Aspergillus, Gibellulopsis*
*Globodera pallida* (Eberlein et al., 2016)	Females	16S – amplicon	*Actinophytocola, Aquabacterium, Bosea, Bradyrhizobium, Brevundimonas, Burkholderia, Dermacoccus, Devosia, Moraxella, Pantoea, Pelomonas, Ralstonia, Rhizobium, Rhodobacter, Sphingopyxis, Streptomyces, Zoogloea, Flavobacteria*
		ITS – amplicon	*Davidiella, Hirsutella, Malassezia, Microdochium, Monographella, Penicillium, Colletotrichum*
*Heterodera schachtii* (Yin et al., 2003a, b)	Cyst	Bacteria – OFRG	Actinobacteria, Cytophaga-Flexibacter-Bacteroides, α-Proteobacteria, β-Proteobacteria, γ-Proteobacteria, *Rhizobium*
		Fungal – OFRG	*Dactylella oviparasitica, Fusarium oxysporum, Lycoperdon*
*Heterodera glycines* (Song et al., 2017)	Cyst	ITS – DGGE	*Geomyces, Aureobasidium, Fusarium, Penicillium, Aspergillus, Cladosporium, Setosphaeria, Alternaria, Mortierella, Cryptococcus, Trichosporon, Galactomyces*
*Heterodera glycines* (Hu et al., 2017)	Cyst	ITS – amplicon	*Trichoderma, Leptosphaeria, Clonostachys, Purpureocillium, Penicillium, Pochonia, Fusarium, Exophiala, Mortierella, Microstroma, Typhula, Phoma, Oudemansiella, Saksenaea, Melanospora, Xylaria, Orbilia, Entoloma*
		16S – amplicon	*Streptomyces, Enterobacter, Acidovorax, Pseudomonas, Variovorax, Rhizobium, Serratia, Massilia, Dactylosporangium, Lentzea, Amycolatopsis, Mesorhizobium, Actinoplanes, Asteroleplasma, Nocardia, Bradyrhizobium, Actinocorallia, Micromonospora, Streptosporangium, Kribbella, Phyllobacterium, Devosia, Nonomuraea, Actinomadura, Aminobacter, Sphingomonas, Shinella, Chitinophaga, Niastella, Steroidobacter, Kineosporia, Luteolibacter, Lysobacter, Rhodanobacter, Echinococcus*
*Heterodera glycines* (Hussain et al., 2018)	Cyst	16S – amplicon	Proteobacteria (*Devosia, Ferrovibrio, Sphingopyxis, Phaselicystis, Sphingomonas, Aquabacterium, Steroidobacter, Lysobacter, Albidiferax, Ideonella, Pseudoduganella, Tahibacter, Bosea, Yersinia, Pseudoxanthomonas, Pseudoduganella, Pseudomonas, Steroidobacter*), Actinobacteria (*Actinophytocola, Actinocorallia, Actinomadura, Nocardia, Planosporangium, Actinoplanes, Promicromonospora, Streptosporangium, Kribbella, Streptomyces, Saccharothrix, Amycolatopsis, Lentzea*), Bacteroidetes (*Taibaiella, Ohtaekwangia, Dyadobacter, Chitinophaga, Candidatus paenicardinium, Niastella), Chlamydiae (Candidatus rhabdochlamydia*), Verrucomicrobia (Verrucomicrobium, Haloferula), Planctomycetes (*Planctomyces*)
*Meloidogyne incognita* (Bent et al., 2008)	Egg masses	Fungal – OFRG	Pochonia, Fusarium, Plectosphaerella, Microdochium, Saccharomyces, Tetracladium, Geomyces, Monacrosporium, Ceratobasidium, Auricularia
*Meloidogyne* spp. (Giné et al., 2016)	Bulk soil	16S – DGGE	*Lysobacter, Flavobacterium, Chryseobacterium, Flexibacter, Steroidobacter, Methylobacterium, Candidatus Solibacter*
		ITS – DGGE	*Pseudaleuria, Fusarium, Preussia, Ctenomyces, Mortierella, Cladosporium, Stachybotrys, Pseudallescheria, Psathyrella, Heydenia*
*Heterodera glycines* (Hamid et al., 2017)	Rhizosphere	ITS – amplicon	*Mortierella, Purpureocillium, Fusarium, Pochonia, Clonostachys, Scleroderma, Penicillium, Aspergillus, Corynespora, Guehomyces, Humicola, Eupenicillium, Cryptococcus, Monographella, Tetracladium, Geomyces, Stachybotrys, Ilyonectria, Myrothecium, Monodictys, Arthrobotrys, Dactylellina, Drechslerella, Haptocillium, Hirsutella, Trichoderma, Acremonium, Penicillium, Nematoctonus, Catenaria*
		16S – amplicon	*Pseudomonas, Massilia, Arthrobacter, Rhizobium, Sphingomonas, Burkholderia, Chitinophaga, Streptomyces, Mesorhizobium, Novosphingobium, Variovorax, Enterobacter, Bradyrhizobium, Bacillus, Niastella, Mucilaginibacter*
*Meloidogyne chitwoodi Pratylenchus neglectus* (Castillo et al., 2017)	Rhizosphere	16S – amplicon	*Pasteuria, Brevibacillus, Bacillus, Pseudomonas, Agrobacterium, Arthrobacter, Burkholderia, Brevundimonas, Clostridium, Corynebacterium, Flavobacterium, Hydrogenophaga, Lysobacter, Methylobacterium, Mycoplana, Phyllobacterium, Rhizobium, Sphingobacterium, Stenotrophomonas, Streptomyces, Variovorax*
*Heterodera glycines* (Hussain et al., 2018)	Rhizosphere	16S – amplicon	Proteobacteria (*Pseudomonas, Ensifer, Shinella, Rhizobium, Aquabacterium*), Firmicutes (*Pasteuria*), Bacteroidetes (*Candidatus paenicardinium*), Verrucomicrobia (*Haloferula*)
*Heterodera glycines* (Hussain et al., 2018)	Root endosphere	16S – amplicon	Proteobacteria (*Phyllobacterium, Aquamicrobium, Rhizobium, Rhodomicrobium, Luteimonas, Cupriavidus, Bdellovibrio, Devosia, Pseudomonas, Aquabacterium, Hydrogenophaga, Shinella, Ensifer, Pseudoxanthomonas, Bosea*), Actinobacteria (*Agromyces, Micromonosporaceae, Lentzea, Streptomyces, Glycomyces, Microbacteriaceae*), Firmicutes (*Pasteuria, Fictibacillus*), Bacteroidetes (*Candidatus paenicardinium*), Planctomycetes (*Planctomyces, Rhodopirellula*)

To get further insights into the active microbiota and to identify other antagonistic microbial traits involved in nematode-suppressive soils, shotgun metagenomic (Chapelle et al., 2016), metatranscriptomic (Chapelle et al., 2016), and metabolomic (Hayden et al., 2019) approaches might be applied or combined. By combining metagenomics and metatranscriptomics, Chapelle et al. (2016) found that upon fungal invasion the stress-related genes were up-regulated in bacterial families inhabiting the rhizosphere of sugar beet grown in a suppressive soil. The fungal pathogen *Rhizoctonia solani* secreted phenylacetic and oxalic acid during plant root colonization, leading to oxidative stress in plants and rhizosphere microbes. This oxidative stress response caused a shift in bacterial composition and activated antagonistic traits that confined pathogen infection. Furthermore, Hayden et al. (2019) using metabolomic and metatranscriptomic analyses revealed that sugar molecules were more abundant in the *R. solani*-suppressive than in *R. solani*-conducive soils. They found that the most abundant compound associated with suppressive soils was the antimicrobial secondary metabolite macrocarpal L. These studies demonstrated that the combination of various approaches could provide us with a detailed understanding of the microbes and mechanisms involved in disease-suppressive soils. To acknowledge the enormous plant contribution in nematode antagonism by beneficial microbes in soil, in the next two sections we will focus on nematode-microbe interactions in the rhizosphere and on the involvement of root exudations in this regard.

## Importance of the Rhizosphere Microbiota in Plant-Nematode Interactions

**FIGURE 2:**
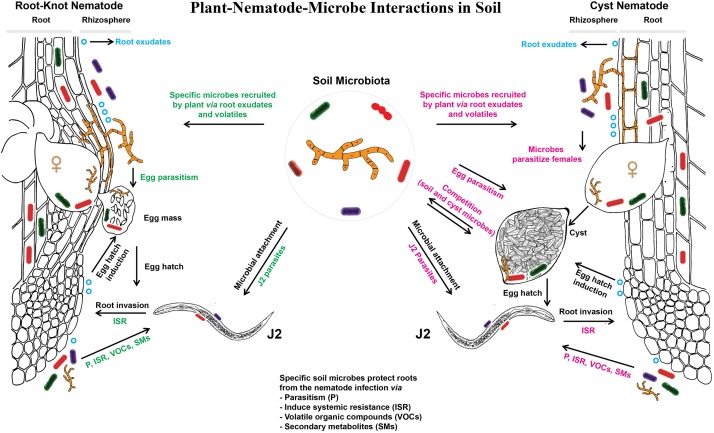
Below-ground communication between plant roots, associated microbiota, and plant-parasitic root-knot or cyst nematodes.

The most intense interactions between the mobile stages of PPN and soil microorganisms occur in the rhizosphere. Nematodes sense environmental signals using different sensilla, but the most studied are paired anterior sensory organs, called amphids. They are positioned in the nematode head region and consist of a glandular sheath cell, a socket cell, and the secretions that are produced by a sheath cell and that surround many dendritic processes (Perry, 1996). Amphids are responsible for navigating PPN to the host roots, providing their positive interaction with the chemical cues released from the roots. Reynolds et al. (2011) proposed that the host range of a certain nematode species determines whether they respond to the general or more specific plant cues. Gene expression in *Pratylenchus coffeae* was influenced, in a host-specific manner, by cell wall components that were either secreted by the plant or released by degradation of root tissue (Bell et al., 2019). Cellulose or xylan from host plants upregulated the level of β-1,4-endoglucanase or β-1,4-endoxylanase genes, respectively. Plants can interfere with PPN signaling (Manohar et al., 2020), and it is likely that also microbial activities modulate and mediate plant-nematode communication. Blocking plant cues or nematode chemoreceptors results in nematode repellence from the roots (Zuckerman, 1983). However, plant root components alone do not entirely affect nematode attractiveness to the roots and a subsequent invasion. In order to reach the roots, nematodes need to travel through a one to several millimeters long soil space in the close vicinity of the root that is called rhizosphere. This is a very active zone along the growing roots with a constant water and nutrient uptake, root exudations and microbial activities (Berg and Smalla, 2009). It was estimated that soil acidity is 10-fold higher in the rhizosphere than in bulk soil, suggesting very pronounced root effects on chemical and biological characteristics of the surrounding soil (Hübel and Beck, 1993). Both soil type and plant genotype contribute to the composition of the microbial communities in the rhizosphere (Micallef et al., 2009). The composition of exudates varies among plant species (Badri and Vivanco, 2009; Eisenhauer et al., 2016) and genotypes (Mönchgesang et al., 2016). The modulation of the microbiome by root exudates induces a feedback of the microbiome. This plant-soil feedback can support growth and/or health of the plant and of the next plants growing in the same soil (van der Putten et al., 2016; Brinkman et al., 2017). Plant-soil feedback effects varied among plant species, but the number of PPN that fed on the roots of a particular plant species correlated with a negative plant-soil feedback (Wilschut et al., 2019). Plants rely on their root microbiome when they are under attack by pathogens and parasites, leading to a selective enrichment of plant-protective microbes and microbial activities in the rhizosphere (Bakker et al., 2018). The importance of the rhizosphere microbiome in plant-nematode interactions has been extensively reviewed (Kerry, 2000; Griffiths et al., 2007; Sikora et al., 2007; Nyaku et al., 2017; Topalović and Heuer, 2019). Importantly, some recent studies have demonstrated that the transfer of the rhizosphere microbiome from one crop to another significantly alleviated nematode infection and enhanced the plant resistance to PPN (Elhady et al., 2018; Zhou et al., 2019). This effect depended on the plant species. The plant’s own microbiome protected it better from root invasion of PPN than the bulk soil microbiome or a foreign microbiome, with the notable exception of the highly suppressive maize microbiome (Elhady et al., 2018). Furthermore, Hussain et al. (2018) revealed that the same subset of microbial OTU was commonly enriched in the rhizosphere and root endosphere upon nematode challenge in suppressive soil. This points out a strong communication between plants and their associated microbiota when under a threat by PPN. In this case, the rhizosphere microbiota can act as a first line of defense against invading PPN, while the root endosphere microbiota can act as a second line of defense against successfully invaded nematodes. Depending on the microorganisms associated with the roots, PPN can be antagonized by parasitism, intoxication, production of volatile organic compounds, or by microbially induced systemic resistance in plants (Figure 2). Most often, the united efforts of more than one mode of action are employed by soil microbiota in nematode suppression. Topalović et al. (2020b) have shown in a split-root experiment that microorganisms from a suppressive soil induced systemic resistance in tomato plants against *M. hapla*, but a combination of a direct antagonism and induced resistance provided a better protection to the plants. They also showed in a sterile system that the microbes attaching to the cuticle of *M. hapla* in the suppressive soil induced systemic resistance in the plant upon nematode invasion (Topalović et al., 2020a). In addition, in recent years scientists have intensively explored the role of plant and microbial volatile organic compounds on nematode parasitism (Huang et al., 2010; Yang et al., 2012; Barros et al., 2014; Xu et al., 2015; Estupiñan-López et al., 2018; Silva et al., 2018; Pedroso et al., 2019). Thus, the potential of root microbiota to assist plants in fighting PPN is enormous. In the next section, the role of root exudates on nematode-microbe interactions is more comprehensively discussed.

## The Role of the Nematode Surface Coat and Root Exudates in Plant-Nematode-Microbe Interactions

When it comes to the interactions with soil microorganisms, the most intriguing feature of the nematode’s body is the SC. This is a carbohydrate-rich protein layer secreted over the epicuticle by the hypodermis (Curtis et al., 2011), or by the excretory and nervous system (Lin and McClure, 1996). The SC is probably involved in recognizing the lectin-like protein molecules on the microbial surface by its glycohydrate epitopes (Bird, 2004; Davies and Curtis, 2011). Bird (2004) produced a very concise and detailed review on the recognition and consequences of nematode-microbe interactions. Depending on the microorganism, the association between the two can result in parasitism, e.g., *Anguina* sp. and a parasitic *Rathayibacter* sp. (Bird, 1985; Riley and Reardon, 1995), or *Meloidogyne* sp. and a parasitic *Pasteuria* sp. (Davies, 2009), commensalism, e.g., *Longidorus* sp., *Xiphinema* sp., *Trichodorus* sp., and *Paratrichodorus* sp. as virus vectors (Taylor and Brown, 1997), mutualism, e.g., *Steinernema* sp. and their endosymbiotic bacteria *Xenorhabdus* sp. (Lacey and Georgis, 2012), or a phoretic association, e.g., *Steinernema* and the bacterium *Paenibacillus* sp. (El-Borai et al., 2005). Some studies have shown that root exudates and some phytohormones, like indole-acetic acid, can alter the SC of PPN (Akhkha et al., 2002; Curtis, 2008) and affect the subsequent attachment of microorganisms to the nematode surface (Singh et al., 2014; Liu et al., 2017). Similarly, López de Mendoza et al. (2000) recorded an increased uptake of the fluorescent lipid analog 5-N-(octadecanoyl) aminofluorescein (AF18) by the SC of J2 of *M. incognita* and the animal parasitic nematode *Haemonchus contortus* after exposure to tomato root exudates for 30 min. Root exudates from different plant species have a variable effect on attachment of endospores of *P. penetrans* to J2 of root-knot nematodes (Singh et al., 2014; Liu et al., 2017). This suggests that a correlation exists between the host type of root exudates and nematode-microbe interactions in soil. It was found that the attachment of *P. penetrans* endospores to J2 of *M. incognita* was increased when J2 were exposed to the root exudates in the presence of soil microorganisms (Singh et al., 2014). As nematode secretions and the SC components are first perceived by the plant when encountering nematodes (Curtis, 2008; Mendy et al., 2017), masking these nematode receptors upon microbial attachment to the nematode in soil might reduce nematode recognition by the plant. On the other hand, some endoparasitic nematodes, like *Meloidogyne* spp., hide from the strong plant defense responses by moving through the apoplast until reaching permanent feeding sites (Sijmons et al., 1991; Williamson and Hussey, 1996; Shah et al., 2017). It was recently shown that microorganisms attaching to the J2 of *M. hapla* in suppressive soil before J2 penetration into the roots increase their recognition by the plant by up-regulating several PTI-responsive defense genes (Topalović et al., 2020a). Studying the microbially induced chemical and metabolic changes in nematode perception by the plant would better elucidate the exact mechanisms in this tripartite interaction. In addition, the nematode SC is subjected to a constant turnover and renewal (Spiegel and McClure, 1995; Spiegel et al., 1995; Gravato-Nobre et al., 1999; Curtis et al., 2011). A study with animal parasitic nematodes showed that the SC changed minutes after nematodes are exposed to the conditions equivalent to those inside the host, excluding the influence of molting in such a short time (Proudfoot et al., 1993). The discarded SC deposits inside the plant or animal host can trick plant immune effectors and keep them away from mobile nematodes that are in search for a more stable feeding site (Blaxter et al., 1992). Thus, although the root exudates are important in nematode attraction to the roots, they can also directly affect nematode interactions with soil microorganisms by inducing changes in the surface of PPN. The nature of these interactions and the type of soil microorganisms and root exudates determine whether PPN will evade the plant immune responses or fail to infect the plant.

## Concluding Remarks and Future Perspectives

Plant-parasitic nematodes cause considerable losses to vegetables and agronomic crops worldwide. Notably, most research has been done on a few species of the endoparasitic sedentary genera *Meloidogyne*, *Heterodera*, and *Globodera*. Investigations on other PPN will give the opportunity to generalize conclusions on the interactions of PPN within the phytobiome and to acknowledge species or genus specific differences in life strategies (Topalović et al., 2020b). Microorganisms contributing to the natural suppression of PPN in soils have been studied using culture-dependent and culture-independent methods. Specific suppression of soils is mainly attributed to the antagonistic activities of selective microbial consortia against eggs, juveniles, or females of PPN. The *in vitro* effects of isolated microbial strains often fail to reproduce upon reintroduction of strains in conducive field soils, due to their inability to colonize roots and fully express putative modes of action. While isolation of specific microbial antagonists is essential for their mass production and application in integrated pest management of PPN, the DNA/RNA-based characterization of the microbiomes associated with plant and nematode is important for a more comprehensive understanding of the interactions between nematodes and their natural enemies in soil, rhizosphere and plant. In parallel, the success of the nematode infection depends on how the signaling molecules from the nematode surface are perceived by the plant roots. Semiochemicals exuded from the plant with root exudates are not only important for the communication between plants and nematodes, but they also directly affect nematode-microbe interactions by modulating components of the nematode surface. Depending on the nematode host range and the microbial composition in soil, endoparasitic nematodes can evade plant defense responses and successfully establish inside the roots, or they can be antagonized inside (root endosphere) or outside (rhizosphere) the plant. Thus, plants infinitely rely on their root microbiome during nematode invasion, leading to the enrichment of a specific subset of plant-protective microbes in the rhizosphere and root endosphere (Hussain et al., 2018). Moreover, a thorough understanding of interkingdom microbe-microbe interactions in soil ecosystems may help to enrich the abundance and activities of indigenous keystone microbial taxa by agricultural management practices such as crop rotation (Hu et al., 2018) or tillage (Hu et al., 2017), or through soil amendments like chitin (Cretoiu et al., 2013) or chitosan (Mwaheb et al., 2017). Unraveling the microbiome structure and functions in nematode-suppressive soils and understanding their relationship with the plant will provide us with more knowledge on the mechanisms responsible for nematode suppression, and may help to develop synthetic microbial communities or manage the soil biome for biocontrol of PPN.

## Author Contributions

OT and MH conceived, developed the theme, and wrote the manuscript. MH prepared figures. HH contributed to ideas and provided critical revisions of the manuscript.

## Conflict of Interest

The authors declare that the research was conducted in the absence of any commercial or financial relationships that could be construed as a potential conflict of interest.
